# Epidemiology and economic impact of bovine cysticercosis and taeniosis caused by *Taenia saginata* in northeastern Spain (Catalonia)

**DOI:** 10.1186/s13071-018-2931-4

**Published:** 2018-06-28

**Authors:** Minerva Laranjo-González, Brecht Devleesschauwer, Famke Jansen, Pierre Dorny, Céline Dupuy, Ana Requena-Méndez, Alberto Allepuz

**Affiliations:** 1 IRTA, Centre de Recerca en Sanitat Animal (CReSA, IRTA-UAB), Campus de la Universitat Autònoma de Barcelona, 08193 Bellaterra, Spain; 2 Department of Public Health and Surveillance, Scientific Institute of Public Health (WIV-ISP), 1200 Brussels, Belgium; 3 Department of Biomedical Sciences, Institute of Tropical Medicine, 155 Nationalestraat, B-2000 Antwerp, Belgium; 4 Laboratory of Parasitology, Faculty of Veterinary Medicine, Ghent University, 133 Salisburylaan, B-9820 Merelbeke, Belgium; 5 General Directorate for Food, Slaughterhouses and Cutting Plants Board, 69401 Lyon, France; 6 Barcelona Institute for Global Health (ISGlobal, Hospital Clínic-Universitat de Barcelona), Barcelona, Spain; 7 Departament de Sanitat i Anatomia Animals, Universitat Autònoma de Barcelona (UAB), 08193 Bellaterra, Barcelona, Spain

**Keywords:** *Taenia saginata*, Bovine cysticercosis, Taeniosis, Economic impact

## Abstract

**Background:**

In Catalonia (north-eastern Spain), *Taenia saginata* has been described in cattle but its occurrence in humans is unclear. Moreover, whether cattle acquired the infection in Catalonia or outside Catalonia and its economic impact have not been investigated. This study aimed to estimate the prevalence and spatial distribution of bovine cysticercosis in Catalonia (2008–2015), and the burden from *T. saginata* upon the animal and human sectors in Catalonia (2013–2015).

**Methods:**

Data on cattle diagnosed with cysticercosis at meat inspection were collected and analysed. Cattle movement history was used to identify the most likely place of bovine cysticercosis infection and to investigate its spatial distribution. Data on taeniosis treatment (niclosamide and praziquantel) costs and their supply in Catalonia as well as data on patients attending primary care with diagnosis of taeniosis were collected. The financial impact associated with *T. saginata* due to carcasses condemned and frozen, meat inspection and human taeniosis was estimated.

**Results:**

During 2008–2015, between 18 and 107 cattle were found positive for cysticercosis each year (prevalence at slaughter of 0.010%). Movement history was available for 44% of the infected cattle and in 53% of them Catalonia was identified as the place where the infection was acquired with highest probability. Two significant bovine cysticercosis clusters were detected. The number of patients diagnosed with taeniosis in primary care during the period 2013–2016 was 41–63/year. The overall economic impact of *T. saginata* (2013–2015) amounted to 154,903 €/year (95% CI: 113,075–196,762). Meat inspection accounted for 81.9% (95% CI: 75.8–86.2%) of the costs, followed by costs due to carcass condemnation and freezing (9.4%; 95% CI: 6.9–12.8%), and taeniosis-associated costs (8.7%; 95% CI: 6.7–11.6%). Costs due to freezing and condemnation of carcasses reached 19,442 €/year (95% CI: 17,528–21,391) (509 €/lightly infected carcass and 1,140 €/heavily infected carcass). Taeniosis-associated costs were estimated at 12,848.5 €/year (237 €/patient).

**Conclusions:**

The public health risk of *T. saginata* in the area seems to be low. The economic impact due to *T. saginata* was mainly attributed to meat inspection. The cost due to carcass condemnation and freezing was limited compared to the revenue of the beef sector. Developing and implementing risk-based surveillance is needed to lower the costs of meat inspection. Considering cattle movements might be useful in the development of such a strategy.

## Background

*Taenia saginata* is a food-borne parasite that infects humans (definitive host) and bovines (intermediate host). Humans acquire the infection (taeniosis) by consuming raw or undercooked beef containing infective cysticerci (*T. saginata* metacestode larval stage). The adult tapeworm develops in the human intestine and produces gravid proglottids that are shed in the stools or leave the anus spontaneously [1]. Bovines acquire the infection (bovine cysticercosis) by accidentally ingesting water, pasture or fodder contaminated with *T. saginata* ova originating from human faeces [2]. Following ingestion, the eggs hatch and release oncospheres that migrate, through the circulatory system, mainly to muscular tissues where they establish and develop into cysticerci. In the muscles they will remain infective for months or even years before undergoing degeneration and calcification [3]. Bovine cysticercosis in naturally infected cattle does not cause clinical signs [4].

The main preventive measure to control *T. saginata* is based on the detection of cysticerci and implementation of sanitary measures during meat inspection. In the European Union (EU), Regulation (EC) No 854/2004 [5] establishes that all bovines over six weeks-old are to be individually examined for bovine cysticercosis through visual examination, incision and palpation of several muscular tissues. Carcasses found to be heavily infected (generalised infection) are to be condemned. However, if the infection is not generalised (light infection), the parts not infected can be declared fit for human consumption after undergoing a cold treatment.

Human taeniosis is generally asymptomatic and easily treated with anthelmintics [2]. However, symptoms such as anal pruritus, abdominal discomfort, weight loss, diarrhoea, nausea, epigastric pain and vomiting have been described [1, 4]. Despite its low impact on public health [6], it is generally assumed that *T. saginata* incurs a high economic impact for the beef sector due to condemnation and downgrading of carcasses [3, 7]. Additionally, resources involved in routine meat inspection are being invested [8]. However, the economic significance of this parasite in European countries is not well known and there are no recent estimates quantifying the economic impact [9–11]. Moreover, the impact on public health is difficult to assess as taeniosis is not notifiable and there are no systematic data collection and reporting systems. The number of taeniosis cases is often estimated from sales figures of niclosamide and praziquantel [12]. In Europe, a risk-based surveillance and control approach is encouraged [8, 13] but current knowledge on the epidemiology and impact of bovine cysticercosis is too limited to guide such an approach [4].

In north-eastern Spain (Catalonia) bovine cysticercosis has been detected every year with a prevalence based on meat inspection ranging between 0.015–0.022% since 2005 and with a clustered distribution of infected farms [14]. Previous analyses have not taken into account the movement of animals and the fact that cattle could have been infected in a different location than the last farm that sent the animals to the slaughterhouse. Therefore, these previous estimates might be useful to assess human exposure to *T. saginata* but might be biased if the interest is to assess the burden of bovine cysticercosis in this region. In addition, there are no published data on the number of cases of taeniosis in humans and there are no estimates of the economic impact of bovine cysticercosis in north-eastern Spain (Catalonia).

The aim of this paper was to assess the epidemiology and burden of the *T. saginata* taeniosis/bovine cysticercosis disease complex in the animal and human sectors in Catalonia. We specifically aimed to (i) estimate the prevalence of bovine cysticercosis in cattle slaughtered in Catalonia between 2008 and 2015; (ii) estimate the prevalence and spatial distribution of bovine cysticercosis in Catalonia between 2008 and 2015 (based on the Catalan farm where cattle most likely became infected); and (iii) calculate the economic burden from *T. saginata* upon the animal and human sectors in Catalonia for the years 2013–2015.

## Methods

### Data collection and analysis

#### Bovine cases

The number of cattle in which cysticerci had been detected (i.e. positive animals) during routine post-mortem inspection at the slaughterhouse (Regulation (EC) No 854/2004) [5], the year of detection and the slaughterhouses reporting cases, were provided by the Public Health Agency of Catalonia. Data on all cattle farms (i.e. geographical coordinates, census and production type), the identification of farms that have sent positive animals for slaughter, together with the individual identification codes of the positive animals (when available) and cattle movement history were provided by the Department of Agriculture, Livestock, Fisheries and Food of the Autonomous Government of Catalonia. The number of cattle slaughtered annually in Catalonia was obtained from “Encuesta de sacrificio de Ganado” [15] published by the Spanish Ministry of Agriculture.

##### Identification of the farm where cattle most likely became infected

The movement history of positive animals was used to identify those farms where cattle could have been most likely infected. Individual identification codes of positive cattle were used to retrieve, from the cattle movements database, their age and the time period during which each animal had been on every farm in their movement history. Details on movements occurring outside Catalonia are not kept in this database and therefore it was not possible to calculate the time spent by positive animals on farms outside our area of study. For those movements, based on the date the animal had left or arrived to Catalonia and their date of birth, we calculated the overall time period that each animal had been outside this area.

For each cattle farm the “probability” that the animal had acquired the infection on it was calculated as follows:

$$ {P}_{ij}=\kern0.5em \frac{\left({T}_{ij}\right)}{\left({Age}_i-42\right)} $$

where i is the individual cattle code; j is the cattle farm code (for farms outside Catalonia the code would be “outside”); P_ij_ is the probability with which an animal “i” had acquired the infection on a location “j”; T_ij_ is the time spent by animal “i” on location “j” (days); Age_i_ is the age of the animal “i” (days).

We assumed that the infection could not have been acquired in the last 6 weeks (i.e. 42 days) before slaughter as it is considered that a cyst develops and becomes readily visible and easily detected during post-mortem inspection six weeks after infection [16, 17]. Therefore, we deducted 42 days from the time spent by the infected animal on the last farm (or farms if the time spent on the last one was lower than 42 days).

For each infected animal, the case farm was defined as the farm in their movement history with the highest *P*_*ij*_ value that was located in Catalonia. For the positive animals for which we did not have the individual cattle code and therefore could not obtain movement data, we assumed that the infection could have been acquired on the last farm sending the animals for slaughter (i.e. case farm). In these cases if the last farm was located out of this region these animals were discarded for further spatial analysis. Bovine cysticercosis cases for which both the movement history and the farm sending animals to slaughter were inaccessible were also discarded for further spatial analysis.

##### Estimation of bovine cysticercosis prevalence

The apparent prevalence of bovine cysticercosis at slaughterhouse level was calculated as the number of positive cases detected during meat inspection divided by the total number of slaughtered animals. The apparent prevalence of bovine cysticercosis acquired in the region of Catalonia was calculated as the number of animals that would have most likely been infected in Catalonia divided by the number of cattle slaughtered in Catalonia not coming from farms outside this region. The specificity (100%) and sensitivity (27% for animals with a low level of infestation [18]) of meat inspection were taken into account to calculate the true prevalence of the disease. Specificity was assumed to be 100% since when doubts about the final diagnosis exist, samples of bovine cysticercosis suspected cases are usually sent to the laboratory for confirmation. The true prevalence was calculated using the following formula [19]:

$$ True\ prevalence=\frac{AP-\left(1- Sp\right)}{1-\left[\left(1-S\mathrm{p}\right)+\left(1- Se\right)\right]}=\frac{AP+ Sp-1}{Se+ Sp-1} $$

where *AP* is the apparent prevalence; *Se* is the sensitivity (ranging from 0 to 1); and *Sp* is the specificity (ranging from 0 to 1).

##### Spatial analysis

A spatial analysis to detect geographical clusters of bovine cysticercosis in Catalonia was performed using the free software SaTScan v.9.4.4 (http://www.satscan.org). We ran a purely spatial analysis for clusters with high rates of bovine cysticercosis cases detected from 2008 to 2015. Based on the exact geographical coordinates of each farm, we used a Bernoulli model in which cattle farms were classified as case/control. Case farms were those cattle farms where the infection could have been acquired with the highest probability (based on previous analysis), while controls were the remaining cattle farms.

Details about the spatial scan statistic can be found in Kulldorf et al. [20]. Briefly, this method generates circular zones of continuously varying radii that range from zero up to a maximum cluster size (50% of the population at risk in our case). For each location and window size, a likelihood ratio test is computed based on the number of observed and expected cases within and outside the circular window and compared with the likelihood under the null hypothesis. Under the null hypothesis the expected number of cases in each area is proportional to its population size. The significance of the clusters is assessed using a Monte Carlo hypothesis test (999 replications). A 5% significance level was established. Results from the spatial scan statistics were represented using the free software QGIS v.2.12.2 [21].

#### Human cases

The number of niclosamide and praziquantel treatments prescribed and distributed in Catalonia to treat taeniosis, was made available from the Spanish Agency of Medicines and Medical Devices (AEMPS) for 2015 and 2016. Data on consultations to primary care of patients who, during the period 2013–2016, had a diagnosis of taeniosis (*T. saginata* or unspecified taeniosis) following the ICD-9-CM (International Classification of Diseases, Ninth Revision, Clinical Modification) codes (i.e. 123.2: “*Taenia saginata* infection”; 123.3: “Taeniasis, unspecified”) were retrieved from the database “Conjunt mínim bàsic de dades d’atenció primària” (CMBD-AP) [22]. The CMBD-AP is a registry managed by the Catalan Health Department that gathers information on the pathology seen by primary health care services classified according to the ICD. Duplicate records (i.e. patients seen more than once with the same diagnosis date) were discarded using the patient identification code. Consultations of the same patient with a different diagnosis date were considered to be different taeniosis cases. The extracted data included a patient identification code, county of residency, the *Taenia* species diagnosed, date of diagnosis and date of consultation. The majority of the cases recorded in CMBD-AP [22] were recorded as *Taenia* spp. cases. We assumed that all of them were *T. saginata* as this is, among the three species causing human taeniosis (*T. saginata*, *T. asiatica* and *T. solium*), the only species endemic in Europe.

#### Assessment of the economic impact of *T. saginata* in Catalonia

We estimated the financial impact associated with *T. saginata* by taking into account three components: (i) costs for the cattle owners due to condemnation and freezing of carcasses (2012–2015); (ii) costs for the official veterinary authorities due to the implementation of meat inspection associated with bovine cysticercosis (2012–2015); and (iii) costs associated with human taeniosis (data for human cases belonged to the period 2013–2016). The overall annual cost due to *T. saginata* in Catalonia was estimated only for the period 2013–2015 which are the years for which data on both bovine cysticercosis and human taeniosis were available. The different parameters used to estimate the economic impact of *T. saginata* are described in Tables 1, 2 and explained below.

**Table 1:** Parameters used to estimate the economic losses attributable to *T. saginata* in Catalonia

	Parameter	Value	Source
Costs for the cattle owner due to condemnation and freezing of carcasses	No. of types of infection		
	Generalised	4	Personal communication (Catalan Public Health Agency)
	Localised	144	
	No. of positive animals per age group		
	8–12 months	44	Personal communication (Agriculture Department)
	12–24 months	82	
	> 24 months	22	
	Average carcass weight (kg) per age (months) category annually		
	2012		[25]
	8–12	232.9	
	12–24	274.9	
	> 24	294.1	
	2013		
	8–12	227.0	
	12–24	273.7	
	> 24	294.9	
	2014		
	8–12	230.1	
	12–24	278.1	
	> 24	295.2	
	2015		
	8–12	225.7	
	12–24	283.3	
	> 24	293.0	
	Average weekly carcass price per age category (€/100kg) annually^a^		
	2012		[26]
	8–12	Normal (μ = 369.1, σ = 4.6)	
	12–24	Normal (μ = 385.7, σ = 9.5)	
	> 24	Normal (μ = 224.4, σ = 18.9)	
	2013		
	8–12	Normal (μ = 369.1, σ = 4.6)	
	12–24	Normal (μ = 396.7, σ = 5.0)	
	> 24	Normal (μ = 227.1, σ = 26.5)	
	2014		
	8–12	Normal (μ = 369.1, σ = 4.6)	
	12–24	Normal (μ = 387.2, σ = 17.6)	
	> 24	Normal (μ = 220.4, σ = 25.7)	
	2015		
	8–12	Normal (μ = 369.1, σ = 4.6)	
	12–24	Normal (μ = 371.8, σ = 5.7)	
	> 24	Normal (μ = 215.6, σ = 21.6)	
	Costs of carcass disposal (€/kg)	0.198	Personal communication (rendering company)
	Loss of value of a frozen carcass (%)	Uniform (min = 48, max = 60)	Expert’s opinion (five slaughterhouses)
Costs for the official veterinary authorities due to the implementation of meat inspection associated with bovine cysticercosis	Time taken by meat inspection official auxiliaries in scenario 1		
	Seconds spent per animal to detect bovine cysticercosis through routine inspection (i.e. animals coming from farms where positive animals have never been detected)	Uniform (min = 20, max = 55)	Expert’s opinion (official veterinary teams)
	Time taken by meat inspection official auxiliaries in scenario 2		
	Seconds spent per animal to detect bovine cysticercosis through detailed inspection (i.e. animals coming from farms where positive animals have been detected at some point in time)	Uniform (min = 110, max = 115)	Expert’s opinion (official veterinary teams)
	Time taken by official veterinarians in scenario 2		
	Seconds spent per animal during supervision/inspection of cattle coming from farms where positive animals have been detected at some point in time	Uniform (min = 60, max = 120)	Expert’s opinion (official veterinary teams)
	Time taken by official veterinarians in scenario 3		
	Hours spent per animal when a positive animal is detected during post-mortem inspection	PERT (min = 0.5, mode = 1.75, max = 3)	Expert’s opinion (official veterinary teams)
	Cost of service of meat inspection official auxiliaries (€/hour)	19	Personal communication (Catalan Public Health Agency)
	Cost of service of official veterinarians (€/hour)	37	Personal communication (Catalan Public Health Agency)
	Cost of anatomo-pathological diagnosis (€/unit) (2012–2015)		
	2012	31	Personal communication (Veterinary Pathology Diagnostic Service, Autonomous University of Barcelona)
	2013	31	
	2014	33.1	
	2015	34.7	
	No. of suspect samples sent for anatomo-pathological examination (2012–2015)		
	2012	31	SESC [24]
	2013	18	
	2014	15	
	2015	14	
Costs associated with human taeniosis	No. of cases with a taeniosis diagnosis in primary care during the period 2013–2016 based on ICD-codes	217	[22]
	No. of medical consultations per patient		
	To primary care	1	Expert’s opinion (medical specialist)
	To a specialist	1	Expert’s opinion (medical specialist)
	Therapeutical options used (%)		
	Niclosamide	60	Unpublished data
	Praziquantel	40	Unpublished data
	Cost of medical consultation (€/unit)		
	Primary care consultation	40	[28]
	Specialist consultation	137	Personal communication (Hospital Clínic de Barcelona)
	Diagnostic tests used (%)		
	Microscopy: concentration techniques for intestinal parasites, helminth eggs and cystic forms	50	See text
	Macroscopy: morphological identification of parasites	50	See text
	Cost of diagnostic test (€/unit)		
	Microscopy: concentration techniques for intestinal parasites, helminth eggs and cystic forms	15.3	[28]
	Macroscopy: morphological identification of parasites	9.8	[28]
	Cost of medical treatment (€/unit)		
	Treatment (niclosamide)	5	Personal communication (AEMPS)
	Treatment (praziquantel)	79.3	Personal communication (AEMPS)
	No. of stool samples tested (per patient)	2	Expert’s opinion (medical specialist)

^a^Carcass price of bovines aged 8–12 months was available only for 2015

**Table 2:** Number of animals inspected (2012–2015)

Year	Scenario 1^a^	Scenario 2^b^
2012	458,042	19,507
2013	462,172	21,066
2014	448,210	22,831
2015	475,487	23,682

^a^Animals coming from farms where positive animals have never been detected

^b^Animals coming from farms where positive animals have been detected at some point in time

##### Model implementation

The models were run using the *mc2d* package [23], implemented in R (R Development Core Team 2008) [24]. Monte Carlo simulations (10,000 and 1001 iterations for modelling uncertainty and variability, respectively) were performed and all non-fixed input parameters were included as uncertain or variable parameters.

The parameters for which the experts provided a minimum and maximum value with no further information on whether a value within that range could occur with a higher or lower probability were modelled as a uniform distribution. This kind of distribution is defined by the minimum and maximum values obtained from the experts and, between those limits, a continuous spectrum of values occurs with the same probability. For the only parameter for which experts provided a range of values and also the most likely value (i.e. “time taken by official veterinarians in scenario 3”) we used a PERT distribution, which is defined by a minimum, most likely and maximum values. The parameters used as fixed values with no distribution were those parameters for which we obtained a unique fixed value from data providers with no further detail on whether these values could vary or not.

##### Costs for the cattle owners due to condemnation and freezing of carcasses

This component was calculated as the sum of the cost of all generalised (i.e. condemned carcasses) and localised infections (i.e. frozen carcasses) detected in Catalan slaughterhouses during 2012–2015. The value of the carcasses was estimated based on the average annual carcass weight [25] and the mean weekly carcass price [26] for the different age categories.

Data on the age of the animals were obtained from the Agriculture Department of the Catalan Government. The age of the positive animals was available in just 26% of the cases (38 out of 148). The age of the remaining cases was estimated based on the age distribution of the positive animals detected between 2008 and 2015 for which the age was accessible (167 out of 382). Positive cattle were classified into three age categories (8–12 months; 12–24 months; and > 24 months). The carcass price and weight assigned to each of these categories was based on the market price and weight for various categories (e.g. bovines aged between 8–12 months, uncastrated males of 12–24 months and female bovines that have calved, other female bovines aged over 12 months), which are freely available on the Agriculture Department’s website [25, 26]. A normal distribution was used in order to take into account the variability of the weekly carcass price for each age category along the year. The mean and standard deviation were calculated based on the average weekly carcass price for each age category each year.

The price (per unit of weight) of carcass disposal was provided by a rendering company and included as a fixed parameter in the model. The cost of carcass disposal was calculated based on the weight of the condemned carcasses. The cost of transporting condemnations from the slaughterhouse to the rendering plant was not included as condemned carcasses are usually transported with other animal by-products that are regularly collected in slaughterhouses.

The percentage of value loss of the frozen carcasses was provided by five slaughterhouses of the region. The costs of handling, transport to freezing facilities, freezing treatment and the weight loss of the carcass after freezing were included in the percentage value loss, together with the meat depreciation, as stated by the experts from the slaughterhouses providing the information. In order to take into account the variability in the answers around value loss given by the five slaughterhouses, we included this parameter in the model as an uncertain parameter by using a uniform distribution.

In the case of localised infections, during the entire period (2012–2015), there was only one carcass part condemned (partial condemnation). As details on the weight, size and value of this part were not available, this was not included in the cost estimate. A total of 31 heads and 116 hearts were also condemned. For comparison with other studies, the losses due to rejected offal, heads and hearts were not included in our overall economic burden analysis.

The cost due to condemnation and freezing of carcasses was calculated as follows for each year:

$$ CO=\kern0.5em \sum \limits_j{GI}_j\ast \left({CC}_j+{ CC D}_j\right)+\sum \limits_j{LI}_j\ast {CC}_j\ast LV $$

where CO is the cost for the cattle owners; j is the indicator of the age category (i.e. 8–12 months; 12–24 months and >24 months); GI is the number of generalised infections for each “j” age category; CC is the value of the carcass for each “j” age category; CCD is the cost of carcass disposal for each “j” age category; LI is the number of localised infections for each “j” age category; LV is the percentage of loss of value of the frozen carcass.

##### Costs for the official veterinary authorities due to the implementation of meat inspection associated with bovine cysticercosis

The costs of meat inspection associated with bovine cysticercosis were calculated taking into account three different scenarios: (i) routine inspection: animals coming from farms where positive animals have never been detected (referred to as scenario 1); (ii) detailed inspection: animals coming from farms where positive animals have been detected at some point in time (referred to as scenario 2); and (iii) detection of a positive case (referred to as scenario 3). In scenario 1, routine meat inspection is conducted by meat inspection official auxiliaries. In scenario 2, official veterinarians also intervene either by supervising meat inspection or conducting meat inspection themselves. Carcasses and predilection sites are inspected more carefully, and extra slicing of the heart is performed resulting in a longer period of time dedicated per animal. In scenario 3, the official veterinarian dedicates time to different activities such as carefully examining the carcass, taking samples to send for confirmatory diagnosis, retaining and sending the carcass to be frozen, preparing official documentation or verifying that the carcass has been frozen.

Time dedicated to meat inspection in the different scenarios was collected from official veterinary teams from three of the biggest cattle slaughterhouses in Catalonia (accounting for 60% of the total number of slaughtered animals). Specifically, we collected information on the time dedicated to the inspection of the heart, masticatory muscles, diaphragm, oesophagus, carcass and tongue per animal inspected. The uncertainty around these times provided by the different veterinary teams was taken into account by using uniform distributions (scenarios 1 and 2). In scenario 3 experts provided a minimum, most likely and maximum value for the time dedicated; a PERT distribution was therefore used.

The cost of the official auxiliary and official veterinary services per hour was provided by the Catalan Public Health Agency. The number of animals coming from farms where positive animals have been detected at some point in time were estimated based on number of animals that these farms send to Catalan slaughterhouses in a year. These data were extracted from cattle movement records provided by the Agriculture Department of the Catalan Government.

The number of suspect samples sent for confirmation was provided by the Catalan Slaughterhouse Support Network [27]. The price of one anatomo-pathological exam was obtained from the Veterinary Pathology Diagnostic Service from the Autonomous University of Barcelona.

The cost attributed to meat inspection was calculated as follows:

$$ {MI}_j={TA}_j\ast CTA\ast {AN}_j+{TOV}_j\ast CTOV\ast {AN}_j+\mathrm{SS}\ast \mathrm{DG} $$

where j is the indicator of the scenario (1 to 3); TA is the time dedicated to meat inspection associated to bovine cysticercosis by meat inspector official auxiliaries in each “j” scenario per animal (for official auxiliaries only scenario 1 and 2 were considered); CTA is the cost of the meat inspector official auxiliaries service by unit of time; AN is the number of animals inspected in each “j” scenario; TOV is the time dedicated to meat inspection associated to bovine cysticercosis by official veterinarians in each “j” scenario per animal (for official veterinarians only scenario 2 and 3 were considered); CTOV is the cost of the official veterinary service by unit of time; SS is the number of bovine cysticercosis suspect samples sent for confirmatory diagnosis; DG is the cost of anatomo-pathological diagnosis.

##### Costs associated with human taeniosis

Human taeniosis-associated costs were estimated using the number of cases diagnosed with taeniosis during 2013–2016 (i.e. ICD-9-CM Codes 123.2: “*Taenia saginata* infection”; 123.3: “Taeniasis, unspecified”) retrieved from the CMBD-AP [22]. Additionally, the following assumptions were made: (i) each patient consulted a primary care physician and a specialist, once each; (ii) for each patient 2 stool samples were tested; (iii) 50% of the samples were tested through macroscopic examination and 50% through microscopy (the proportion of cases in which proglottids are found is unknown therefore it was assumed that in half of the cases proglottids would be available for macroscopic examination); (iv) all patients were treated; (v) patients were treated only once; and (vi) 60% of the cases were treated with niclosamide and 40% with praziquantel. These last data were obtained from a questionnaire sent to seven hospital pharmacies of Catalonia in which the most frequent therapeutic option used to treat taeniosis was asked (unpublished data).

The costs of a medical consultation to primary care and to a specialist were obtained from the Catalan Health Service [28] and the Clinic Hospital of Barcelona (personal communication), respectively. The cost of the diagnostic tests was obtained from the Catalan Health Service [28]. The price of niclosamide and praziquantel was made available from the AEMPS (personal communication).

Accordingly, the cost associated with human taeniosis (HT) was calculated as follows:

$$ HT=\kern0.5em NC\ast \left( CVP+ CVE+ DGI+ DGA\right)+0.6\ast NC\ast CN+0.4\ast NC\ast CP $$

where NC is the number of cases; CVP is the cost of a medical consultation by a primary care doctor; CVE is the cost of a medical consultation by a specialist; DGI is the cost of the microscopic parasitological examination; DGA is the cost of the macroscopic parasitological examination; CN is the cost of niclosamide; CP is the cost of praziquantel.

##### Costs not considered in our analysis

Other specific costs not considered in our analysis include outbreak investigations, measures taken at farm level (i.e. changing filters in the water supply system or parasitological controls of farm staff), training for meat inspectors, research projects, costs associated with transport to obtain diagnosis and treatment or opportunity costs associated with obtaining health care. Complications associated to *T. saginata* taeniosis such as appendicitis or gastrointestinal perforations have been described occasionally [29]. As these conditions are very rare any possible costs related to them (e.g. hospitalisation) have not been considered in the analysis.

## Results

### Bovine cases

#### Prevalence of bovine cysticercosis in cattle slaughtered in Catalonia (2008–2015)

The number of positive animals detected in Catalan slaughterhouses between 2008 and 2015 is shown in Table 3. The apparent prevalence detected at slaughterhouse was low (0.010%) and ranged between 0.004–0.022%. Taking into account the low sensitivity of meat inspection the true prevalence was estimated at 0.037%, ranging between 0.014–0.080%.

**Table 3:** Cattle diagnosed by meat inspection with bovine cysticercosis in slaughterhouses in Catalonia (2008–2015)

Year	Positive animals	Animals slaughtered in Catalonia	Apparent prevalence (%)	True prevalence (%)
2008	107	492,678	0.022	0.080
2009	62	473,842	0.013	0.048
2010	40	480,685	0.008	0.031
2011	25	477,388	0.005	0.019
2012	67	477,549	0.014	0.052
2013	18	483,238	0.004	0.014
2014	19	471,041	0.004	0.015
2015	44	499,169	0.009	0.033
Total	382	3,855,590	0.010	0.037

#### Prevalence and spatial distribution of bovine cysticercosis most likely acquired in Catalonia between 2008 and 2015

##### Farm where cattle most likely became infected

Movement history could be retrieved and analysed for 167 cattle, out of a total of 382 meat inspection positives, for which individual identification was available. Based on the probability with which each positive animal acquired the infection on each location of their movement history, 53% (i.e. 88 out of 167) most likely became infected in Catalonia. Out of these, the infection was certainly acquired on a Catalan farm in 21 cattle (13% of the positives) as they never left Catalonia.

In 47% of the cases (79 out of 167) the infection was most likely acquired outside the study area, and 62 out of these (37%) definitely acquired the infection outside as they came to Catalonia only to be slaughtered. Out of the 79 animals that would have acquired the infection outside, in 63 cases the infection would have taken place in other parts of Spain, in 10 cases in other EU countries (1 in Belgium, 8 in France and 1 in Romania) and for 6 the location of the case farm was unknown.

The 88 animals that most likely acquired the infection in Catalonia had been on average on two farms in Catalonia during their lifetime (range of 1–4 farms). The average time that each animal spent on each farm was highly variable depending on the type of farm. While in assembly centres the animals stayed on average 3 days, in production farms they stayed on average 419 days (median of 247 days and a range of 3–4955 days). Of note, in 84% of these cases (74 out of 88 animals) the farm identified as the most likely place of infection was also the last farm sending the animals to slaughter.

Information on the number of times that a farm sent at least one positive animal for slaughter was available for 311 out of the total 382 positive cattle detected. During 2008–2015 the majority (88%) of the farms sending at least one positive animal to slaughter sent positive animals only once, 11% of the farms sent positive batches between 2 and 3 times, and one farm sent positive animals on eight different occasions.

##### Prevalence of bovine cysticercosis in cattle coming from Catalan farms and that were most likely infected in Catalonia

When taking into account only cattle that did not come from farms located outside Catalonia and the cases that had been most likely infected in this area, the apparent prevalence of bovine cysticercosis by meat inspection between 2008 and 2015 was 0.007% and ranged between 0.003–0.015% (Table 4). The calculated true prevalence was 0.025% (range of 0.009–0.054%).

**Table 4:** Cattle diagnosed by meat inspection with bovine cysticercosis that were most likely infected in Catalonia (2008–2015)

Year	Animals infected in Catalonia	Slaughtered animals coming from Catalan herds	Apparent prevalence (%)	True prevalence (%)
2008	54	364,582	0.015	0.054
2009	31	350,643	0.009	0.033
2010	20	355,707	0.006	0.021
2011	13	353,267	0.004	0.013
2012	34	353,386	0.009	0.035
2013	9	357,596	0.003	0.009
2014	10	348,570	0.003	0.010
2015	22	369,385	0.006	0.022
Total	191	2,853,137	0.007	0.025

##### Spatial distribution of bovine cysticercosis in Catalonian farms

**Fig. 1:**
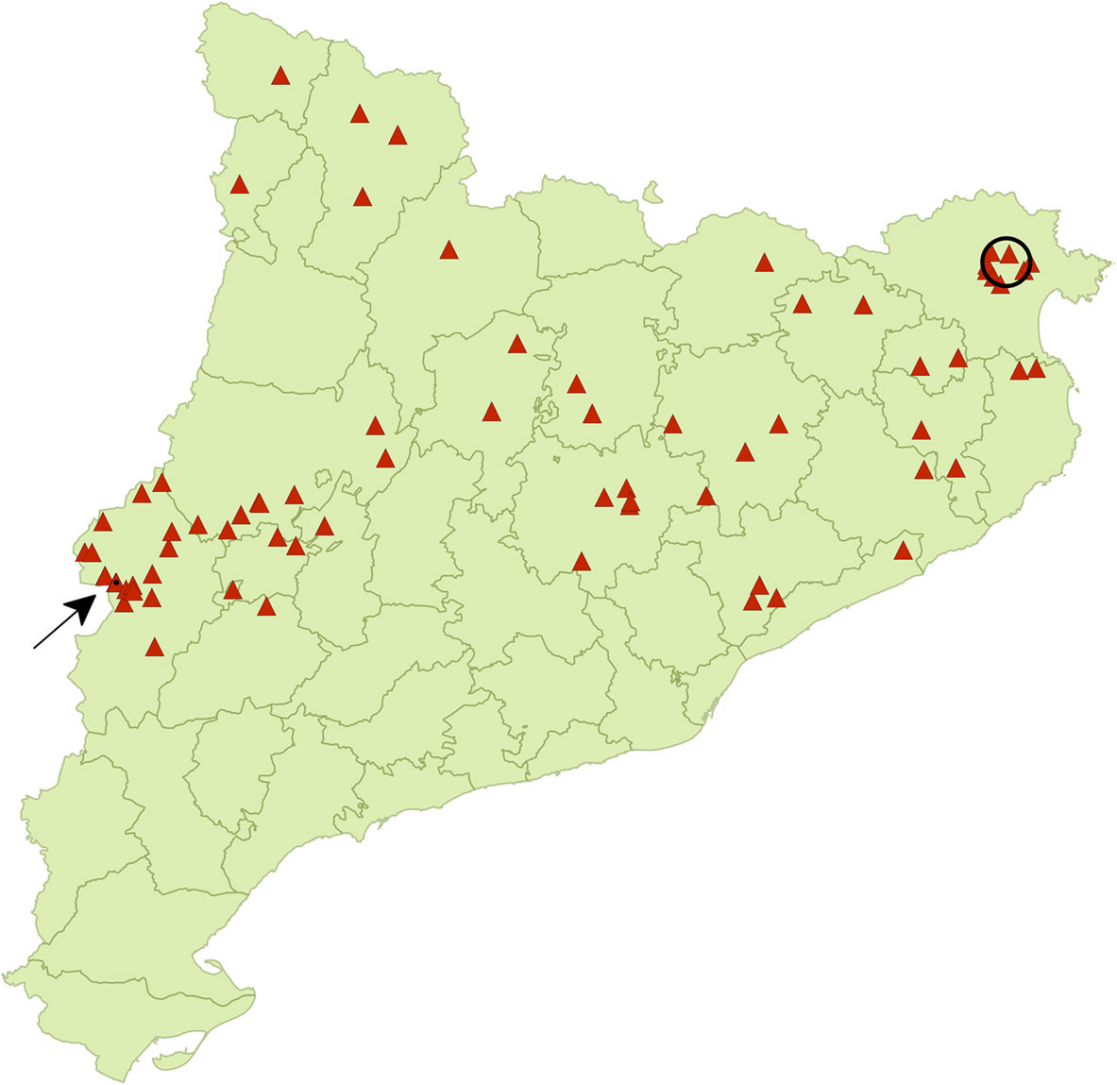
Spatial distribution of significant high rate clusters of bovine cysticercosis identified using a Bernoulli model with a maximum scanning window of 50% of the population at risk (2008–2015). Triangles, case farms; circle, first cluster; arrow, second cluster

The spatial analysis identified two significant clusters of bovine cysticercosis (Fig. 1). The largest cluster was located in the north-east of Catalonia and had a radius of 5.74 km and a relative risk (RR) of 12.8. It comprised 52 farms and had eight observed case farms vs 0.70 expected. The case farms were 7 fattening herds and one beef breeding herd and involved 22 positive animals (1–6 per farm). The mean age of the infected cattle (unknown in two cases) was 1.2 years (range of 9.3 months to 3.3 years). These positive cattle had been detected at slaughter at different points in time from the end of 2008 until the end of 2011 (11 cases at the end of 2008, 5 at the beginning of 2010 and 6 from mid to end of 2011). One of these farms had also sent two positive animals to the slaughterhouse 1.5 years before (May 2007). One other farm also sent positive animals on 3 different occasions during 2007. However, these cases were not included in the spatial analysis as the study period included only cases from 2008 to 2015.

A second cluster, located in the west of the study area, had a radius of 0.17 km and a RR of 58.2. It involved four herds (3 cases *vs* 0.054 expected). All three case farms were dedicated to fattening. The total number of positive animals was three (one per farm) and had been detected at different points in time from the beginning of 2008 to mid-2009. The age of the infected cattle (unknown in one case) was around one year-old.

### Human cases

The number of patients treated for taeniosis in Catalonia (using either niclosamide or praziquantel) was 22 in 2015 and 19 in 2016 (Table 5). Based on the consultations recorded in the CMBD-AP database the number of cases attending primary health care diagnosed with taeniosis during 2013–2016 was 217 (41–63 /year) (Table 5).

**Table 5:** Number of patients attending primary care with diagnosis of taeniosis (2013–2016) and number of taeniosis cases treated with niclosamide and praziquantel (2015–2016) in Catalonia

Year	Taeniosis cases seen at primary health care			Taeniosis cases treated with niclosamide or praziquantel		
	*T. saginata*	*Taenia* spp.	Total	Niclosamide	Praziquantel	Total
2013	2	39	41	na	na	na
2014	0	63	63	na	na	na
2015	1	61	62	6	16	22
2016	0	51	51	9	10	19
Total	3	214	217	15	26	41

*Abbreviation*: *na* not available

### Assessment of the economic impact of *T. saginata* in Catalonia

**Fig. 2:**
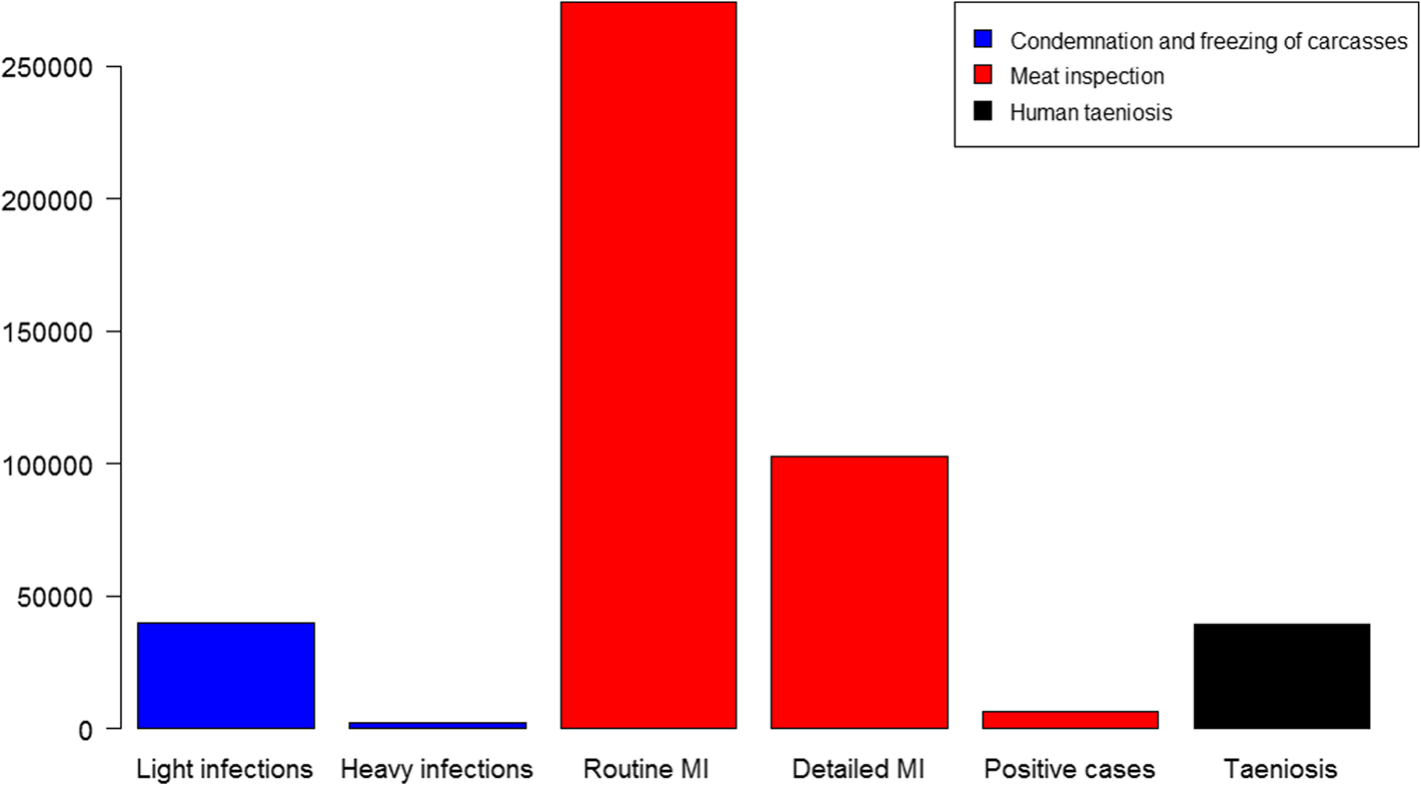
Average costs (€) of the different components associated to *T. saginata* during the period 2013–2015. *Abbreviations*: MI, meat inspection

The overall annual mean economic impact of *T. saginata* in Catalonia during the period 2013–2015 amounted to 154,903 € (95% CI: 113,075–196,762 €). The costs of the different components during the period 2013–2015 are shown in Fig. 2. The major contribution was attributed to the surveillance of bovine cysticercosis at the slaughterhouse as it accounted for 81.9% (95% CI: 75.8–86.2%) of the total costs. The cost for the beef sector due to condemnation and freezing of carcasses was responsible for 9.4% (95% CI: 6.9–12.8%) while the costs associated to human taeniosis accounted for 8.7% (95% CI: 6.7–11.6%) of the total economic impact.

The cost of meat inspection targeting bovine cysticercosis (2012–2015) (mean of 127,566 €/year, 95% CI: 85,818–169,203) (Table 6) was estimated at 0.2 € (95% CI: 0.1–0.3 €) per animal inspected through routine meat inspection, at 1.5 € (95% CI: 1.2–1.8 €) per animal inspected through a detailed meat inspection (originating from farms that have sent positive animals to slaughter at some point in time), and at 99 € (95% CI: 66.3–131.5 €) for the procedures following the detection of a positive.

**Table 6:** Costs (€) for the Official Veterinary services due to meat inspection targeting bovine cysticercosis

Year	MI, routine		MI, detailed		MI, detection of positive carcasses	
	Mean	95% CI	Mean	95% CI	Mean	95% CI
2012	90,903	50,658–130,827	29,624	23,916–35,350	5323	3129–7524
2013	91,723	51,115–132,007	31,992	25,827–38,176	1744	1155–2335
2014	88,952	49,571–128,019	34,672	27,991–41,374	1711	1089–2335
2015	94,365	52,587–135,810	35,965	29,035–42,916	3290	1849–4735

*Abbreviations*: *MI* meat inspection, *CI* confidence interval

The cost due to condemnation and freezing of carcasses (2012–2015), reached a mean of 19,442 €/year (95% CI: 17,528–21,391) (Table 7). Costs due to lightly infected carcasses amounted to 18,301 €/year (95% CI: 16,388–20,250), corresponding to 509 € (95% CI: 455–563 €) per lightly infected carcass. Costs due to heavily infected carcasses (including value loss and disposal cost) were estimated at 1140 €/year (95% CI: 1089–1191), which corresponded to 1140 € (95% CI: 1089–1193 €) per heavily infected carcass; the disposal costs amounted only to 52.2 €/carcass. Considering average prices provided by experts, the value of rejected heads (31) and hearts (116) during the study period amounted to just 358 € (95% CI: 347–369 €).

**Table 7:** Costs (€) for the beef sector due to freezing and condemnation of infected carcasses

Year	Generalised infections (value loss and disposal costs)		Localised infections (value loss)	
	Mean	95% CI	Mean	95% CI
2012	1996	1937–2058	33,161	29,694–36,692
2013	0	0	9261	8293–10,247
2014	2565	2363–2747	8715	7804–9643
2015	0	0	22,068	19,761–24,418

The costs associated to taeniosis were estimated at 12,848.5 €/year corresponding to 236.8 € per patient (25.1 € for diagnosis, 177 € for medical consultations and 34.7 € for treatment).

## Discussion

Previous research conducted on *T. saginata* in north-eastern Spain (Catalonia) [14, 30] focussed only on bovine cysticercosis; the current study therefore provides a more complete picture of the burden of the *T. saginata* taeniosis/bovine cysticercosis complex in this region. This approach is in line with the One Health concept (http://www.onehealthinitiative.com) which promotes an interdisciplinary approach to tackle diseases. Previous research [30] found a seroprevalence of bovine cysticercosis using an antigen ELISA about 50 times higher than the prevalence obtained by visual inspection. However, the public health risk derived from not detecting all the infected carcasses was unclear due to the lack of available data on human taeniosis at that moment. The results of this study suggest that the public health risk might be low as the number of taeniosis cases diagnosed in primary care ranged between just 41 and 63 per year. Surprisingly, the number of taeniosis cases estimated from the supply of niclosamide and praziquantel was even lower (19–22/year). In Spain these drugs cannot be supplied and have to be requested through the Spanish Medicines Agency and prescribed by a specialist. Therefore the number of niclosamide and praziquantel treatments requested and supplied to treat taeniosis could be indicative of the number of taeniosis cases. The difference between the number of cases diagnosed and treated could be due to the use of a different anthelmintic despite the fact that niclosamide and praziquantel are the most frequently used drugs to treat taeniosis [31–33]. The main strength of using the CMBD-AP dataset [22] to retrieve the number of taeniosis cases is the fact that it is an exhaustive compilation of all the primary care activity provided by the Catalan Health System which covers a population of around 7,500,000 [34]. One limitation is the fact that taeniosis is not a notifiable disease and it could be possible that not all taeniosis cases were properly registered. The results of our study contrast with what has been reported in other countries. For example, in Belgium, around 11,000 taeniosis cases have been estimated to occur annually [35]. These differences in the human health impact might be related to differences in the prevalence of bovine cysticercosis. Indeed, in 2013, Belgium reported a prevalence in cattle of 0.12% [36] whereas in Catalonia it was much lower (i.e. 0.004%). Such differences could be partially attributed to different culinary habits, production systems and climate. Risk factors for bovine cysticercosis infection that have been reported include having access to pastures, to risky water sources or to contaminated feed [9]. In Catalonia, most of the animals are kept indoors and therefore, they may be less exposed to *T. saginata* eggs in the environment. In addition, annual precipitation in Catalonia is lower than in countries like Belgium, which may lead to a shorter egg survival time. In our study, it was not known whether the taeniosis cases were acquired from infected animals not detected at meat inspection or imported from elsewhere in Spain or abroad. The place where the taeniosis infection is acquired is normally unknown. Consequently, it is difficult to know if this also plays an important role in the difference between taeniosis prevalence estimated in different countries.

Reported prevalences of bovine cysticercosis are usually based on meat inspection and it is rarely specified whether the cases are autochthonous or not [37]. Our results indicated that half of the affected animals most likely acquired the infection outside the study area. Therefore the true prevalence of bovine cysticercosis in Catalonia, based on cattle not coming from farms outside Catalonia and on the cases that most likely acquired the infection in Catalonia, would be slightly lower (around 0.025% between 2008–2015) than the true prevalence based on all the cases detected in all cattle slaughtered in Catalan slaughterhouses (around 0.037%). Despite some limitations (i.e. movement history not being accessible for all positive cases) the spatial analysis identified two areas with a higher risk of infection taking into account the farm where cattle most likely became infected. The presence of disease clusters has also been reported in studies performed in France and Italy [17, 37]. Disease clusters could be explained by an epidemiological link between farms. Unfortunately, we did not have results of any epidemiological investigation. Other factors involved could be a higher risk of exposure to *T. saginata* eggs through pastures, water or feed in these areas or direct contamination from human tapeworm carriers (e.g. farm workers). Furthermore, research in these areas might be desirable in order to elucidate the chain of infection and try to adopt preventive measures to reduce the risk of infection.

Recent publications highlight the usefulness of implementing risk-based surveillance in areas with low prevalence of bovine cysticercosis [18, 38, 39]. It has been proposed that information on risk factors (e.g. grazing practices in the herd, location of the herd or gender) could be provided, as food chain information, by the farmer prior to slaughter [40], to identify high- and low-risk herds (or animals) [39, 41]. Our results showed that in the majority of the cases, similar to that observed by Dupuy et al. [17] in France, the infection occurred on the last farm before slaughter, but in some cases the infection could have occurred on a different farm. Therefore, the fact that not all animals may become infected on the last farm should be taken into account if a risk-based surveillance is to be implemented in the future. In line with this, based on a study conducted in the UK, Marshall et al. [42] also concluded that cattle movement history could be used to support a more targeted meat inspection strategy.

The assessment of the economic impact revealed that the highest cost associated with *T. saginata* was due to meat inspection (82% of the cost). In Catalonia, a detailed meat inspection of animals originating from farms that have sent positive animals to slaughter at some point in time is performed. The total cost incurred by routine meat inspection (i.e. inspection of animals originating from farms that have not previously sent positive animals to slaughter) was higher than the detailed meat inspection. However, the cost per animal was higher for detailed meat inspection (1.5 €) than for routine meat inspection (0.20 €). Taking into account that most farms sent positive animals to slaughter only once, and that the infection seems not to always occur on the last farm prior to slaughter, not performing detailed meat inspection in the way it is currently performed could reduce the economic cost without losing sensitivity on the surveillance of the disease.

Calculating the costs of meat inspection associated with bovine cysticercosis was challenging. This was due to the fact that the meat inspectors also perform procedures targeting other diseases (e.g. tuberculosis) [5]. To address this, we asked for the time dedicated exclusively to searching and applying sanitary measures related to bovine cysticercosis, but obviously the uncertainty around this estimate is high. Despite that, the time dedicated to routine meat inspection addressing bovine cysticercosis was very similar to what has been found in a similar study performed in Belgium [35].

Overall, the annual costs for the bovine meat sector in Catalonia due to *T. saginata* were not high compared to the revenue generated by the Catalan beef sector (e.g. revenue generated by 124,500 tons of beef that were produced in 2015) [43]. Compared to the costs estimated in other countries (437,730 € in 2016 in mainland France [44] and 3,579,335 €/year in Belgium [35]), the costs in Catalonia were much lower. Nevertheless, these figures are not directly comparable as they are influenced by the prevalence and number of animals slaughtered. In the case of Belgium, costs also included an insurance paid to cover the losses due to bovine cysticercosis that does not exist in Catalonia. Without including insurance costs, the costs per carcass (including value loss and disposal costs) were similar: 509 € and 1140 € per lightly and heavily infected carcasses, respectively, in Catalonia, *versus* an average of 586 € and 998 € per lightly and heavily infected carcasses, respectively, in Belgium [35]. These recent estimates are higher than costs estimated in earlier studies. According to Murrell (1991) [10] losses in industrialised countries amounted to 234 US$ per infected carcass and in England they reached up to £100 per infected carcass [11]. However, caution should be taken when comparing costs between countries and years due to differences in price levels or differences in factors included in the analysis.

In the present study we might have underestimated some costs for the meat sector. For example, the preventive immobilization of a suspect case, until laboratorial results are available, may incur losses for commercial reasons that are difficult to quantify. Additionally, according to experts, when a carcass is frozen it is difficult to find a client willing to buy it and there might be the need to leave it in a freezing room for up to several months. If the carcass cannot be sold, the majority of it will be used for meat preparations (e.g. burgers) resulting in extra costs due to processing.

The costs associated with taeniosis were estimated at 236.8 € per patient, including medical consultation, diagnosis and treatment. In Belgium, these costs were lower ranging between 6.29 € and 72.4 € per patient depending on whether patients consulted a physician or not [35]. In our study, the costs were estimated based on the patients consulting primary care but the number of cases could be underreported as it is not a notifiable disease. In the USA, older estimates of treatment costs (111 US$/patient) [10] were higher than in the present study (34.7 €/patient), but it is not specified whether medical consultations and diagnosis were accounted for in these estimates.

Our estimates of the taeniosis-associated costs are only approximate due to several limitations. When estimating this component we assumed that all the taeniosis cases registered in the CMBD-AP had been treated with praziquantel or niclosamide. However according to the AEMPS the number of cases treated with these drugs per year was lower. It could be possible that an extra 30–40 cases/year diagnosed but not treated with these anthelmintics were treated with another treatment regime. However, we do not know which other treatment could have been used, the number of doses prescribed or the price of this other therapy. Additionally, it could also be possible that some of the extra cases registered in the CMBD-AP were recorded as taeniosis cases as a result of miscoding. These types of errors have been reported to occur when using ICD coding systems. In the same way, it might have been possible that some taeniosis cases had not been registered as such in the database, especially when it is not a notifiable disease. Overall we believe that these limitations do not have a major impact on the results due to the very low number of cases being diagnosed each year.

## Conclusions

Through this study we believe to provide a relatively complete picture of the *T. saginata* taeniosis/bovine cysticercosis disease complex in north-eastern Spain. The public health risk derived from failing to detect every bovine cysticercosis-infected carcass seems to be low in the area of study as there were a very low number of taeniosis cases. The economic impact associated with *T. saginata* was mainly attributed to meat inspection and borne by the public veterinary services. The cost for the beef sector was much lower and relatively limited compared to the revenue generated by the sector. The cost for the public veterinary services might be reduced through some changes in the surveillance of this disease and further efforts in this direction might be desirable. Possible changes could include the suppression of the detailed meat inspection and the development of a risk-based surveillance strategy. The identification of the most likely farm where cattle became infected shows that animal movements need to be taken into account in the development of such strategy.

## Abbreviations

AEMPS: Spanish Agency of Medicines and Medical Devices
CMBD-AP: “Conjunt mínim bàsic de dades d'atenció primària”
CYSTINET: European Network on Taeniosis/Cysticercosis
EC: European Commission
ICD: International Classification of Diseases
ICD-9-CM: International Classification of Diseases, Ninth Revision, Clinical Modification
